# An Overview of Shiraz Emergency Medical Services, Dispatch to Treatment

**DOI:** 10.5812/ircmj.10982

**Published:** 2013-09-05

**Authors:** Mahmoudreza Peyravi, Per Örtenwal, Ahmadreza Djalali, Amir Khorram-Manesh

**Affiliations:** 1 Prehospital and Disaster Medicine Centre, Sahlgrenska Academy, Gothenburg University, Gothenburg, Sweden; 2 Medical Schools, Shiraz University of Medical Sciences, Shiraz, IR Iran; 3 Center for Research and Education in Emergency and Disaster Medicine, Università degli Studi del Piemonte Orientale Novara, Italy

**Keywords:** Emergency Medical Service, Communication Systems, Reaction Time, Triage

## Abstract

**Background:**

Advanced ambulance service (Emergency Medical Services/EMS) is considered to be an integral part of emergency medical care as the first assets responding to emergencies and disasters in the prehospital setting in most developed countries.

**Objectives:**

The aim of this study was to evaluate the current situation of Shiraz’s EMS by comparing data obtained during two different time periods.

**Materials and Methods:**

This is a retrospective analytic and comparative study in which data obtained from Shiraz EMS during two one-year periods (21st of March 2011 to 20th of March 2012 and 22nd of September 1999 to 21st of September 2000) were compared. Furthermore, these data were also compared with available data from Gothenburg’s EMS (2010).

**Results:**

Of 84084 missions performed by Shiraz EMS during one year trauma cases were the most common [39282 (46.7%)]. The most common cause of trauma was road traffic accidents (RTA) (27257; 76.5%). Near 56% of all patients were transported to hospitals; some 47% by ambulances and 8.8% by private cars. Around 36.2% of patients received definitive medical treatment at the scene. While there was an increase in response and evacuation times, the number of deaths at scene before ambulance arrival decreased.

**Conclusions:**

Although Shiraz’s EMS has expanded during last decade and the mortality rate at scene has decreased, the number of RTA-related trauma cases, along with the response and evacuation time, has increased. More than one third of the patients received definitive treatment and could be dismissed directly from the scene. Standardized triage and treatment protocols are needed to improve the EMS activity.

## 1. Background

Every day 16000 people die worldwide due to injuries, of which around 20% are due to Road Traffic Accidents (RTA) (1). Without effective preventive measures, fatalities and injuries related to trauma would be increased up to 65% between the years 2000 to 2020 throughout the world (2). According to the World Health Organization (WHO) over 90% of the fatalities occur in low and middle income countries (1, 3). Reducing the mortality rate is the greatest challenge for public health organizations. Thus, proper preventive systems, management of human and physical resources, and designing new strategy plans are among the most important concerns to achieve this goal (1-3). Rapid urbanization and an increasing number of motor vehicles in developing countries such as Iran result in increased mortality and morbidity due to trauma (4). The most common cause of death in Iran, after cardiovascular diseases, is trauma related to RTA (5, 6).

Emergency Medical Services (EMS) is the first line of encounter in the management of casualties (7). The prehospital response is an important factor for defining prognosis of all injured patients. In this regard, appropriate response and short time intervals (response time, scene time and evacuation time) together with adequate care are the most important aims for EMS (7-10). These systems are also the backbone of disaster response and should build such preparedness on their daily activities (11, 12). The impact of time intervals has been discussed in many studies, published during last decades (13-16). Iranian EMS was established in Iran in 1975 in close cooperation with American EMS. It was organized as a governmental organization with services free of charge for all patients. Since then it has improved and expanded and become a nation-wide organization with a responsibility also to act during major incidents and disasters. However, similar to other developing countries, it is faced with infrastructural problems such as traffic congestion, narrow roads, and some organizational issues such as lack of human resources and short-comings in command and control systems (17). Each ambulance is staffed with one nurse with expanded training in anesthesiology and one paramedic educated in Basic Life Support (six months training). Moreover, one ambulance is designed to work as mobile ICU (Intensive Care Unit) and is staffed with one general practitioner (GP), one nurse and one paramedic as mentioned above. Another GP works as consultant for technicians at dispatch center to make medical decisions in dubious cases. Besides national EMS, some private ambulance companies transfer nonurgent patients. Looking at improvement as measurable parameters, the response time, for a normal call, was set to less than eight minutes in cities and less than 15 minutes in suburban areas. This setting has been met in some cities, while it has not been achieved in larger urban areas yet (18). Shiraz is located in the southern part of Iran, northwest of Fars Province and is one of the five largest urban areas in Iran with 1.7 million inhabitants and 340 km2 in size (19).

## 2. Objectives

The aim of this study was to give a comprehensive overview of the current situation in Shiraz for EMS activities, including; response, scene, and evacuation times of missions and also its patient’s characteristics such as vital signs and Glasgow Coma Scale (GCS). In addition, we also evaluated the outcome of this review by comparing the results obtained from this study to data collected during 1999-2000 as well as data from a western EMS.

## 3. Materials and Methods

### 3.1. Data Collection

This is a retrospective analytic and comparative study in which, all ambulance missions by Shiraz EMS during two one-year periods were included. The study population consists of all missions irrespective of age and gender. All mission data are registered in a database at Shiraz EMS dispatch center; this was performed manually before 2008 and digitally since then. It is a part of a national data registry in which the following parameters can be found; age, gender, chief complaint, type and result of a mission, etc. All data are recorded by the officer in charge at the dispatch center after termination of each mission. Completed data for each mission are kept in the original registry at each ambulance station. The date and time intervals (response time, scene time, evacuation time and total time) for each mission are recorded automatically.

1) Data collection 2011-2012

The data was collected between 21st of March 2011 and 20th of March 2012, and included all missions during this time period. The data was obtained digitally from the national data registry.

2) Data collection 1999-2000

The data was collected between 22nd of September 1999 and 21st of September 2000. The data during this time period was recorded manually. For this study the data was chosen randomly from all missions performed on the 5th, 15th and 25th of each month.

### 3.2. Ethical Permission

This study was approved by the ethical committee of Shiraz Medical University (2011-100/7 Feb.2011).

### 3.3. Statistics

Data analysis was conducted using SPSS version 15 (SPSS Inc., Chicago, Illinois, USA). The data analysis is expressed using descriptive statistics including range, and mean ± Standard Deviation (SD). Frequency and percentage of categorical data are presented.

## 4. Results

During the recent study period (2011-2012), Shiraz’s EMS performed 84084 missions divided into following categories; trauma 39282 (46.7%), neurology 21936 (26%), cardiovascular 10889 (13%), airway problems 4782 (5.7%), psychiatry 1710 (2%), intoxications 1342 (1.6%), surgery 1272 (1.5%), internal medicine 304 (0.4%), infections 40 (0.01%), others 378 (0.5%). In 2149 cases the diagnosis was not available (2.6%). In 65062 cases the gender of the patients could be determined [male 40487 (62.3%) and female 24575 (37.7%)].

Table 1 shows the vital parameters recorded by the ambulance crew when examining the patients. In 80% of missions the patients were visited by a medic. Thus blood pressure, pulse rate, respiratory rate and Glasgow Coma Scale (GCS) could be evaluated. In missing cases, no evaluations were performed due to human error or the fact that patients were transported to the hospitals or back home privately before ambulances arrived. The zero figures represent cases in which no value for blood pressure, pulse rate or respiratory rate was detected. The mean ± SD pulse rate was 82 ± 13, and 4.7 percent of patients (2941) had tachycardia (pulse rate > 120). The mean ± SD for respiratory rate was 17 ± 3 and near .5% of casualties (277) had tachypnea (respiratory rate > 29). GCS ranged between 3 and 15. In 1.5% it was ≤ 8, in 0.3% it was 9 - 11, in 1% it was 12 - 13 and in 97.2% it was ≥ 14. Table 2 demonstrates the available data (mean time ± SD and median) for response times (time from receiving calls until ambulance arrives at the scene of the accident), the on-scene time (time spent at the scene), evacuation time (time taking the patient or injured to hospital) and total time. 

**Table 1.** Vital Signs and GCS Registered During 2011 - 2012

Variable	Number	Minimum	Maximum	Mean ± SD
**Systolic Blood Pressure**	58929	00	300	120.92 ± 21.30
**Diastolic Blood Pressure**	52438	00	190	77.19 ± 10.71
**Respiratory Rate**	61930	00	52	17.36 ± 2.89
**Pulse Rate**	61430	00	224	82.55 ± 13.14
**Glasgow Coma Scale**	54804	3	15	14.80 ± 1.10

**Table 2.** Time Intervals Registered During 2011 - 2012

Variable	Number	Minimum	Maximum	Mean ± SD (Median)
**Response time (minute)**	68021	1	134.28	10.50 ± 5.04 (9.53)
**Scene time (minute)**	70465	1	152.28	16.64 ± 10.26 (14.98)
**Evacuation time (minute)**	34730	1	143.95	19.41 ± 12.41 (17.15)
**Total time (minute)**	76805	1	166.36	42.33 ± 25.74 (37.63)

Table 3 shows the mechanism of injury divided into various groups. The most common cause of trauma was RTA (27257; 76.5%) in both urban and suburban areas, followed by fall and violence. The rate of accidents increased 4.5% from spring to summer and decreased to 5.5% from autumn to winter, but only for traffic accident in urban areas. The main causes for transportation were registered for 46091 patients (54.8%). In remaining cases transportation was performed privately, and no information was available. Decreased level of consciousness was the most common reason (13.8%) for ambulance request, followed by chest pain in 10.5% of cases, dyspnea in 5.7%, and convulsion (epilepsy) in 4.7% of cases. 

**Table 3.** Type of Traumas in Patients Registered During 2011 - 2012

Variable	Number	Percent
**Accident in urban area**	24528	68.9
**Accident in countryside**	2729	7.6
**Violence**	2853	8.0
**Fall**	4810	13.5
**Trauma during pregnancy**	258	0.7
**Trauma during work**	51	0.1
**Bite**	58	0.2
**Trauma with electricity**	166	0.5
**Foreign body in airway**	78	0.2
**Drowning**	28	0.1
**Collapse landslide**	39	0.1
**Eye trauma**	19	0.1
**Total**	35629	100

Of 84084 (100%) patients, around 56% were transported to hospitals. Some 47% were transferred by ambulances and 8.8% by private cars. Around 36.2% of patients received definitive medical treatment at the scene (minor injuries, nonemergency cases and those refusing to be transported to hospitals) and were discharged from the scene directly. Also, 4.6 % of accidents did not have any injured at all. The number of deaths at scene, before ambulance arrival, was 2.5%, of whom 0.3% (198) was due to RTA. Twenty-two persons died after arrival of the ambulance crew; 15 died at the scene, while 7 persons died during transport to the hospital. Table 4 shows a six-fold increase in daily missions during 2011-2012 compared to data from 1999-2000. There is a more frequent use of ambulances in ages 21-40 years, highly dominated in men. There is also an increasing number of trauma cases. The response, scene and evacuation times, remain largely the same. Less people are managed at scene, while more people are using private cars to transfer patients to the hospitals. The number of deaths before ambulance arrival has decreased. 

**Table 4.** Compare Indicators of Shiraz Emergency Medical Services Between Periods of 12 Years

Indicator	1999 - 2000	2011 - 2012
**Patients characteristics**		
Mean of daily mission	42	230
Male	44.7%	62.3%
Female	46.9%	37.7%
Mean age	45.8 ± 22.3	41.07 ± 21.54
Age group 1 - 20	16.2%	15.1%
Age group 21 - 40	28.8	40.3%
Age group 41 - 60	25%	23.3%
Age group up 60	30%	20.3%
**Chief complains**		
Chest pain	23.5%	19%
Neurology and psychology	15.8%	28%
Trauma	19.4%	46.7%
**Locations**		
Incident Inside the city	99.6%	92.4%
Incident outside the city	0.4%	7.6%
**Time intervals, min**		
Response time	8.9 ± 4.7	10.50 ± 5.04
On-scene time	17.6 ± 9.4	16.64 ± 10.26
Evacuation time	16.3 ± 8.7	19.41 ± 12.41
Total time	42.6 ± 13.8	42.33 ± 25.74
**Result**		
Transfer to hospital	49.7%	47%
Definitive treatment at scene	41.8%	36.2%
Transfer with private vehicles / patient refusal	3.6%	8.8%
Dead before arrival of ambulance	4.1%	2.5%
Cancelation, wrong address	0.8%	1.1%
Available ambulances / day	7 - 9	31
**Availability**		
Available personnel / day (ambulance staff)	14 - 18	62 + 1 Doctor in mobile ICU = 63
Mean of missions for each ambulance / day	5.3	7.3

## 5. Discussion

Different factors influence the outcome of prehospital management of patients with critical conditions. One such factor is training and education (20). Besides increasing the number of ambulances and staff, new skills and continuing training programs are necessities for an effective and sufficient EMS. New guidelines and strategies have been implemented in Shiraz during the last decades (18, 20, 21). However, the growing numbers of inhabitants as well as motor vehicles are challenging factors in Shiraz, as well as other large cities in other developing countries (22). In this study, the comparison between two time periods (12 years apart) indicated a 2.4 fold increase in the number of trauma cases attended by EMS. Almost all traumas were related to RTA. This can be partly explained by increasing number of vehicles and urbanization in Shiraz, but probably also by an increased utilization of the EMS by public in general, but in trauma cases particularly.

The number of deaths on the scene decreased from 4.1% in the year 2000 to 2.5% in 2012. Explanations for this reduction could include the introduction of better driving education and also promotion of infrastructures for road and vehicle safety (23, 24). To be prepared also emphasizes the need for a shorter response time, which depends on a chain of reactions and decisions-making starting from dispatch center, involving triage and valid guidelines (25). As an example, while Shiraz EMS uses a two-scale triage system (emergency run or not urgent), the Swedish city Gothenburg’s EMS uses a four-scale triage method when dispatching an ambulance. To the best of our knowledge, there is no standardized or validated triage guideline at dispatch centers in Shiraz (Iran). This would be a challenge in Iran to effectively use the EMS.

Although there is a decrease in scene time 2012, in comparison to that of 1999-2000 year data (Table 3), the response and evacuation times have slightly increased. Though not significant, the trend of elongated response times may be due to 1) increasing demand for EMS services and 2) traffic congestion. Other studies have also pointed out factors such as inadequate public education and location of patient and the distance from the emergency department as main reasons for longer response time ( 13 , 25 ). As demonstrated, another 8.8% of patients were transferred to hospitals by private cars. Taking this figure into consideration, the response time would be higher than presented if all patients were to be transported by ambulances. Lack of confidence in availability of ambulances and/or in staff’s skills, may be another reason why people use private cars to transport patients to hospitals; an important issue for future EMS authorities in Shiraz to deal with. 

This study also reveals that 36.2% of patients were not transported to the hospital and were either not in the need of medical attention or received definitive treatment at the scene, since Shiraz EMS has the authority to refuse a transport from the scene. As a comparison, all patients requesting ambulance transport in Gothenburg, Sweden, are transported to hospital and the ambulance crew has no right to refuse a transport. This in turn would lead to insufficient use of ambulances and jeopardizes the regional preparedness for major incidents and disasters (26). Although Shiraz’s healthcare may avoid these problems by discharging patients directly from the scene, the quality of care should be investigated by identifying whether these patients get the correct diagnosis and adequate treatment.

**Figure 1.**
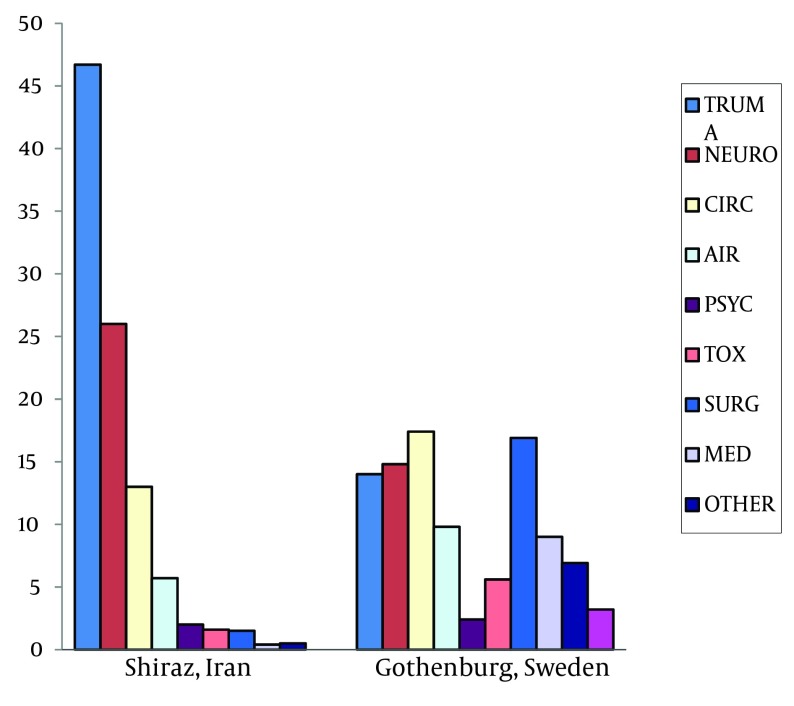
Causes of Transportation: a Comparison Between Shiraz and Gothenburg

Registered vital signs at the scene demonstrates the severity of cases. However, they also imply the necessity of creating a new protocol to define the priorities of patients at scene and during the transportation ( 17 ). New triage methods such as Medical Emergency Triage and Treatment System (METTS), which are used widely in Sweden can be one measure to improve the patient care at the prehospital and hospital levels ( 27 ). Our data also shows that trauma and neurologic cases are more common as causes of transportation in Shiraz than Gothenburg (Figure 1). In Gothenburg, however, the number of transported patients with other diagnosis; cardiovascular symptoms, airway symptoms, intoxications, infectious diseases, etc. are much more common than Shiraz ( 25 ). One reason for this difference might be the lack of validated guidelines or the use of private cars for transportation. Another reason can be differences in traffic regulations and driving habits. 

### 5.1. Policy Implication

There must be a quality control in the system to evaluate some of the medical measures taken by EMS such as treating and discharging the patients from the scene. A new guideline for dispatching and a common triage system would also increase the quality and credibility of EMS. These measures would increase the quality of given healthcare and elevate the credibility of the system and eliminate the need for private transportation and probably lead to improved survival rates.

This study emphasizes the necessity of common guidelines within the whole chain of emergency medical care, both prehospital and hospital. It also implies the need for continuous education and training, besides better planning and higher availability of EMS.
